# Emergency department attendances and GP patient satisfaction

**DOI:** 10.1080/17571472.2017.1333616

**Published:** 2017-06-05

**Authors:** Keith Hurst, Deirdre Kelley-Patterson, Andy Knapton

**Affiliations:** a Hurst Research Ltd., Nottinghamshire, UK; b Centre for the Study of Policy and Practice in Health and Social Care, University of West London, Brentford, UK; c Strategic Modelling Analysis and Planning Limited (SMAP), Winchester, UK

**Keywords:** Workforce planning and development, GP patient satisfaction, ED attendances

## Abstract

**Background:**

Despite invaluable national data, reasons for the relentless rise in England’s emergency department (ED) attendances remain elusive.

**Setting:**

All EDs and general practices in England.

**Question:**

Are rising ED attendances related to general practice patient satisfaction, i.e. if patients are unable to get a convenient appointment with their general practitioner (GP), then do they attend their local ED for diagnosis, treatment and care instead?

**Method:**

GP patient satisfaction and ED attendance data were extracted from national data warehouses and organised into two groups: (i) England clinical commissioning group (CCG) areas and (ii) a London CCG subset. Data from London CCGs were compared with CCGs outside London.

**Results:**

ED attendances were strongly correlated with GP patient satisfaction data in non-London CCGs, e.g. if patients said they had difficulty obtaining a convenient appointment at their general practice, then local ED attendances increased. Associations were repeated when other GP perception data were explored, e.g. if patients were satisfied with GPs and practice nurses, then they were less likely to attend their local EDs. However, these associations were not found in the London CCG subset despite lower satisfaction with London GP services.

**Discussion and Conclusions:**

Although our study generates valuable insights into ED attendances, the reasons why London general practice patient and ED attendance data don’t show the same associations found outside London warrants further study. Diverting patients from EDs to primary care services may not be straight forward as many would like to believe.

## Why this matters to us

In a recent publication in this journal (whole system quality: local benchmarking to improve workforce planning), we argued that openly available strategic data-sets enable primary care managers and practitioners to demonstrate many system relationships. In our second case study, also using data from publicly available databases [1,2]_,_ we explore new workforce planning and development relationships; i.e. associations between failing primary care services and emergency department (ED) attendances: important evidence for primary care community managers and practitioners arguing for additional resources or different working styles. In London, as elsewhere in England, NHS leaders have argued that better and more localised care by general practitioners (GPs) will take pressure off London’s EDs. In this case study, we question whether this solution for London is as straightforward as many would like to believe.

## Key message

Diverting patients from emergency departments (EDs) to primary care services may not be straight forward.

## Case study

In 2015–2016:

(1) 20.5 million (m) ED patient attendances were recorded in NHS England.
(2) April to December attendances increased 2.01%: from 14.9 m in 2014–2015 to 15.2 m in 2015–2016.
(3) Winter hit ED staff the hardest; i.e. attendances between January and March 2016 increased 12.2% compared to the same period in 2015.
(4) There were:

• 7.6 m (37.1%) ED attendances resulting in discharge with no follow-up;
• 4.1 m (20%), which led to hospital admission;
• 4 m (19.5%) were discharged for follow-up by GPs; and
• 2.6 m (12.7%) were referred to out-patient services [2].

(5) Daily average ED attendance in 106 English NHS Trusts was 341 (standard deviation (sd) = 199) [2]:

• 45% were classified as Level 1 (routine care).
• 41% were Level 2 (minimum care).
• 11% were Level 3 (constant, but not continuous care).
• 3% were Level 4 (at least one-to-one care).

Clearly, ED staff look after many sick patients (i.e. 48 Level 3 and 4 patients daily), but could some or all Level 1 (routine care) patients receive diagnoses, treatment and care from alternative provision such as general practitioner (GP) or pharmacy services, so that ED workloads can at least be stabilised and possibly reduced?

## Patient satisfaction with GP services

What causes the relentless increase in ED attendances, especially among the 45% Level 1 patients who may not be emergencies? Could dissatisfaction with GP services drive patients to attend EDs? If yes, then are London healthcare services different to those elsewhere in England? In this case study, we answer these questions by exploring relationships between GP patient satisfaction and ED attendances. We compare London GP and ED performance with their England counterparts. We hypothesise that patients dissatisfied with GP services will attend EDs for diagnosis, treatment and care. If our hypothesis is supported, then simply educating and encouraging prospective ED patients to seek alternative care may not be enough.

We extracted 11 key GP patient perception data-sets from the NHS Benchmarking Database [2] (Table 1).

**Table 1.** GP patient perception data.

Source	England	London	
CCGs	179	32	
*Data*-*set*	*Patient response* (%)		*p* =
1. Getting a GP appointment was easy	25	23	0.101
2. Surgery opens at convenient times	74	70	0.0001
3. Almost always manage to see my preferred GP	36	31	0.0001
4. There weren’t any appointments for the day I wanted	48	51	0.0008
5. I was satisfied with the OoH service when my surgery was closed	30	24	0.0001
6. I Went to ED when my surgery was closed	33	40	0.0001
7. The way my GP listened to me was good	51	44	0.0001
8. The way the practice nurse listened to me was good	46	37	0.0001
9. I have full confidence in my GP	64	56	0.0001
10. I have full confidence in the general practice nurse	62	50	0.0001
11. Overall experience with my GP service is good	43	36	0.0001

Note: Key: *p*, probability.

Table 1 shows that London-based general practices consistently underperform on 11 measures (Rows 1–11) compared to practices elsewhere in England. But are the differences statistically significant? The data in Table 1 are non-parametric, so we used the Wilcoxon–Mann–Whitney statistical test to compare London and England GP patient satisfaction data. We aggregated data at clinical commissioning group (CCG) level because practice level patient satisfaction survey response rates, in some areas, were too low for a meaningful analysis; i.e. only 17% of patients in some general practices completed their patient satisfaction questionnaire [2].

Table 1 shows that differences between England and London CCGs were statistically significant in all 11 data-sets except ‘1. Getting an appointment was easy’. That is, the differences between London general practices and those outside London are highly significant and could not have occurred through chance alone. London general practice patients, therefore, are less satisfied with important general practice structures and processes, such as their consultation.

Extending our study by hypothesising that general practice patients are more likely to attend their local ED owing to negative general practice service perceptions (Table 1, Row 11) and specific impressions (Rows 1–10), therefore, is at least reasonable and probably essential to help solve the rising ED attendance challenge.

## Association between GP patient satisfaction and ED attendances

We tested our GP patient satisfaction and ED attendance hypothesis by correlating ED attendances with 10 GP service measures. We brought forward measures 1–10 from Table 1 and paired them with local ED attendance data, which are summarised in Table 2. We found that GP patient satisfaction scores in a CCG catchment area were correlated with attendances at EDs serving the same patient population. So we separated GP patient satisfaction and corresponding ED attendance data into two groups: (i) England CCGs; and (ii) and London CCGs.

**Table 2.** GP patient perception and ED attendance: correlations.

*Data*-*set*	England	London
	*r*_*s*_ =	*r*_*s*_ =
1. Getting a GP appointment was easy	−0.122*	−0.061
2. Surgery opens at convenient times.	−0.157*	−0.115
3. Almost always manage to see my preferred GP	−0.274*	−0.001
4. There weren’t any appointments for the day I wanted	0.211*	0.038
5. I was satisfied with OoH service when my surgery was closed	−0.278*	−0.053
6. I Went to ED when my surgery was closed	0.142*	0.170
7. The way my GP listened to me was good	−0.309*	−0.091
8. The way the general practice nurse listened to me was good	−0.217*	−0.112
9. I have full confidence in my GP	−0.336*	−0.09
10. I have full confidence in the general practice nurse	−0.251*	−0.157

Note: Key: *significant at the *p* <= 0.0008 level.

Our ED attendance and GP perception data weren’t normally distributed, so we used Spearman’s non-parametric correlation (*r*_*s*_) to test relationships between GP patient satisfaction and ED attendances. The England *r*_*s*_ values in Table 2 range from −0.336 to 0.211:

• An *r*_*s*_ approaching 1 indicates a positive correlation; i.e. as one variable rises so does the other; e.g. patient satisfaction increases as consultation time with a GP rises.
• An *r*_*s*_ nearing −1 indicates a negative correlation; i.e. as one variable rises the other falls; e.g. positive perceptions about general practice services are related to falling ED attendances.
• If *r*_*s*_ = 0, then there is no association; i.e. data pairs fluctuate randomly.

Table 2, Row 4 (unsurprisingly) shows that English ED attendances increase if GP appointments weren’t convenient for patients and when general practices were closed (Row 6). Table 2, on the other hand, suggests that ED attendances throughout England fall when:

• It’s easy to obtain a GP appointment (Row 1).
• When general practices open at convenient times (Row 2).
• When patients manage to see their preferred GPs (Row 3).
• When patients were satisfied with out-of-hours services (Row 5).
• When patients sense that doctors and nurses listen to them (Rows 7 and 8).
• When patients are confident in their doctors and nurses (Rows 9 and 10).

Table 2 show the associations between general practice patient perceptions and ED attendances are all highly significant in England; i.e. appointment systems; opening hours; clinician–patient interaction; and confidence in professionals. These are unlikely to be due to chance alone. Although following the same trends as England, correlations between London patient perceptions and London ED attendance weren’t statistically significant (Table 2). Therefore, we can’t accept our hypothesis that London patients, dissatisfied with GP services, are more likely to attend EDs for diagnosis, treatment and care, even though London patients were more negative about their general practice services than their counterparts elsewhere in England.

One possible explanation for the different perceived effect on ED attendances in London and England (appointment systems; opening hours; clinician–patient interaction; and confidence in professionals in Table 2) is that London EDs are concentrated and patients can access convenient transport compared to patients in rural England. Patients living on London CCG borders may have a choice of EDs, so general practice patient satisfaction data may be crossing CCG boundaries and ED staff are serving patients who may be shopping around, and who may not consistently attend the same ED. ‘Shopping around’ data aren’t collected in the NHS; clearly, an important research and development topic.

## Discussion

Correlations are associations between data-sets; i.e. they do not signify cause and effect. Even when associations between GP patient satisfaction and ED attendances are strong, we can’t be sure that patients can be persuaded to seek primary care services based on improving GP service alone, especially in London. Nevertheless, Table 1 indicates that London GPs may need to improve their services and raise them at least to service quality levels found elsewhere in England if London EDs are to receive some respite.

Table 1 also reveals significant positive patient perceptions. For example, almosTrusts into ED special measures if staff perform poot three quarters of the patients responding to GP patient satisfaction surveys felt that their surgery opening hours were convenient (Row 2) and half could get a suitable appointment (Row 4) in primary care services facing rising demands. Confidence in general practice clinical staff was relatively high (Rows 9 and 10). These elements are strong foundations on which to build.

Alternatives to general practice services aren’t well explored in the literature; i.e. we don’t know whether pharmacy customer perceptions are negative or positive in a general practice context, so there may be merit in exploring what patients think about or have experience with pharmacy staff who act as alternatives to general practice services. There are other variables that influence ED attendances, which are worth exploring. For example, the four-hour ED wait target (the standard that 95% of all ED patients should be discharged, admitted or transferred within four hours) may encourage patients to attend EDs rather than their local surgeries, knowing that they have access to full diagnostic investigations and treatments in the ED. Is it possible that the ED four-hour target may incentivise patients to attend? This issue gains importance because NHS regulators suggest placing Trusts into ED special measures if staff perform poorly on the four-hour wait standard [3]_._ Reducing ED pressures by initiating a corresponding general practice waiting time target to incentivise patients to attend their general practices will need significant investment by the government.

Reviewing routinely gathered data in this way has potential to evaluate, in real-time, complex interventions – when many things happen at the same time, building from local strengths. For example, the government announced in March 2017 budget statement that £100 m will be spent employing GPs in EDs (http://www.bbc.co.uk/news/uk-politics-39203784). Providers may decide to use these GPs differently; e.g. integrating with Out-of-Hours services and Community Hubs. Routinely gathered data could help to evaluate such natural experiments. Furthermore, we could gather data beyond patients’ service perceptions, which could reveal the extent to which healthcare practitioners and patients understand the whole system and local initiatives that facilitate self-help and collaborative care.

## Conflict of interest

All three authors deliver commercial workforce planning education and training courses commissioned by HEE for primary care organisations working across West, North, Central and East London. Keith Hurst develops and maintains the NHS Benchmarking Database (cited in the case study), which is used to support several training and education programmes delivered by University of West London staff.

## Disclosure statement

No potential conflict of interest was reported by the authors.
